# CinE caRdiac magneTic resonAnce to predIct veNTricular arrhYthmia (CERTAINTY)

**DOI:** 10.1038/s41598-021-02111-7

**Published:** 2021-11-22

**Authors:** Julian Krebs, Tommaso Mansi, Hervé Delingette, Bin Lou, Joao A. C. Lima, Susumu Tao, Luisa A. Ciuffo, Sanaz Norgard, Barbara Butcher, Wei H. Lee, Ela Chamera, Timm-Michael Dickfeld, Michael Stillabower, Joseph E. Marine, Robert G. Weiss, Gordon F. Tomaselli, Henry Halperin, Katherine C. Wu, Hiroshi Ashikaga

**Affiliations:** 1 Digital Technology and Innovation Division, Siemens Healthineers, Princeton, NJ USA; 2 Université Côte d’Azur, Inria, Epione Team, Sophia Antipolis, France; 3 Division of Cardiology, Department of Medicine, Johns Hopkins University School of Medicine, 600 N Wolfe Street, Carnegie 568, Baltimore, MD 21287 USA; 4 The Russell H. Morgan Department of Radiology and Radiological Science, Johns Hopkins University School of Medicine, Baltimore, MD USA; 5 University of Maryland School of Medicine, Baltimore, MD USA; 6 Christiana Care Health Systems Inc., Newark, DE USA; 7 Albert Einstein College of Medicine, Bronx, NY USA

**Keywords:** Ventricular fibrillation, Ventricular tachycardia, Cardiac device therapy

## Abstract

Better models to identify individuals at low risk of ventricular arrhythmia (VA) are needed for implantable cardioverter-defibrillator (ICD) candidates to mitigate the risk of ICD-related complications. We designed the CERTAINTY study (CinE caRdiac magneTic resonAnce to predIct veNTricular arrhYthmia) with deep learning for VA risk prediction from cine cardiac magnetic resonance (CMR). Using a training cohort of primary prevention ICD recipients (n = 350, 97 women, median age 59 years, 178 ischemic cardiomyopathy) who underwent CMR immediately prior to ICD implantation, we developed two neural networks: *Cine Fingerprint Extractor* and *Risk Predictor*. The former extracts cardiac structure and function features from cine CMR in a form of cine fingerprint in a fully unsupervised fashion, and the latter takes in the cine fingerprint and outputs disease outcomes as a cine risk score. Patients with VA (n = 96) had a significantly higher cine risk score than those without VA. Multivariate analysis showed that the cine risk score was significantly associated with VA after adjusting for clinical characteristics, cardiac structure and function including CMR-derived scar extent. These findings indicate that non-contrast, cine CMR inherently contains features to improve VA risk prediction in primary prevention ICD candidates. We solicit participation from multiple centers for external validation.

## Introduction

Implantable cardioverter-defibrillator (ICD) therapy has become the cornerstone for the primary prevention of sudden cardiac death (SCD) in patients with systolic heart failure (HF) and reduced left ventricular ejection fraction (LVEF)^1^ due to both ischemic (ICM) and nonischemic cardiomyopathy (NICM). Although the survival benefit of primary prevention ICD is incontrovertible, the rate of appropriate ICD therapies due to ventricular arrhythmia (VA) is relatively low at 1.1–5.1% per year^2^. In contrast, the adverse event rate exceeds the rate of appropriate therapies in individuals at low risk for SCD. For example, device infection rates are 1.4–2.0% per year^3^, and rates of inappropriate shocks approach 5–20% per year^4^, which are associated with increased mortality^5^ and decreased quality of life^6^. In addition, LVEF improvement occurs in up to 25–50% of patients which correlates with diminished SCD risk due to VA^7^. Thus, LVEF is far from being a comprehensive imaging feature to predict VA. Recently, other imaging features of cardiac structure and function were found to be independent predictors of VA, including the extent of heterogeneous myocardial tissue (‘gray zone’) on late gadolinium enhancement (LGE) cardiac magnetic resonance (CMR)^8^, and right ventricular (RV)^9^ and left atrial (LA) function^10^.

Artificial intelligence (AI) algorithms based on deep learning consist of learning complex models directly from data sets. Initial success of AI applications in medical imaging was demonstrated by confirming expert-level diagnoses^11^. Recently, AI has been shown to predict personalized prognosis, such as individual responses to lung cancer therapy^12^ and survival for patients with pulmonary hypertension^13^. While traditional machine learning approaches rely on handcrafted, previously recognized features extracted from medical images, AI can also automatically generate a patient-specific fingerprint containing inherent features of cardiac structure and function from cine CMR^14^ in an unsupervised fashion^15^.

The CERTAINTY study (CinE caRdiac magneTic resonAnce to predIct veNTricular arrhythmia) utilizes deep learning for VA risk prediction for individual patients from non-contrast cine CMR images in primary prevention ICD candidates. The findings from the CERTAINTY study are expected to improve our understanding of the mechanisms that predispose to VA, with the hope to develop a new paradigm to identify high- and low-risk individuals by extracting features associated with increased VA risk from cine CMR images in an unsupervised fashion. This article presents an overview of the CERTAINTY design, a descriptive analysis of the demographics of the study cohort, deep learning network architecture, results within the training cohort, and solicitation of participation to contribute external validation data sets.

## Results

### Study population

The inclusion and exclusion criteria of the CERTAINTY study population are described in Table 1^1^. Baseline characteristics of the training cohort (n = 350) by VA occurrence are summarized in Table 2. The median age was 59 years, and 97 patients (28%) were female. The etiology of HF was ischemic heart disease in 178 patients (51%), and cardiac resynchronization therapy with an ICD (CRT-D) was implanted in 100 patients (29%). The median baseline LVEF was 26%. After a median follow-up of 7.1 years, the primary endpoint was observed in 96 patients (incidence rate of 4.57 per 100 person-years, Table 3). Thirty five patients (10%) received appropriate antitachycardia pacing (ATP) without appropriate ICD shock, and the remaining patients received appropriate ICD shocks. Patients with the primary endpoint (n = 96) were more likely to be male; had larger LV size, extent of total LV LGE, and larger LA size; and lower LA total emptying function than patients without VA events (n = 254).

**Table 1:** CERTAINTY inclusion/exclusion criteria.

Inclusion criteria	Exclusion criteria
18 to 80 years of age	ICD implantation for secondary prevention
Cardiac magnetic resonance (CMR) with intravenous gadolinium contrast within 30 days prior to ICD/CRT implantation	Patients with a permanent pacemaker or a preexisting class 1 indication for pacemaker implantation
History of acute myocardial infarction ≥ 40 days old (confirmed by persistent pathologic Q waves on ECG, clinical reports of CPK-MB > 3 times the upper limit of normal, or a fixed perfusion defect on nuclear imaging) with an ejection fraction (EF) ≤ 30% and no history of revascularization within the last 3 months	Patients with New York Heart Association Class IV heart failure (unless undergoing CRT implantation)
History of ischemic or nonischemic left ventricular systolic dysfunction with stable NYHA Class II to III heart failure symptoms for ≥ 3 months on optimal pharmacotherapy and an EF ≤ 35%. For CRT patients, EF ≤ 35%, QRS > 120 ms, NYHA Class III to IV heart failure symptoms on optimal pharmacotherapy	Patients with history of a confirmed myocardial within 40 days of implant or revascularization within the last 3 months
	Patients fulfilling class III indications for primary prevention ICD implantation

**Table 2:** Baseline characteristics.

	Total (n = 350)	Ventricular arrhythmia (n = 96)	No ventricular arrhythmia (n = 254)	p value
**Demographics**				
Age, year	59 (50, 67)	58 (49, 65)	59 (50, 68)	0.868
Female, n (%)	97 (28%)	18 (19%)	79 (31%)	0.021
Body surface area, m^2^	2.00 (1.83, 2.16)	2.00 (1.87, 2.20)	2.00 (1.82, 2.15)	0.241
Follow-up duration, years	7.1 (4.3, 10.0)	5.0 (2.4, 7.9)	6.7 (4.1, 9.7)	< 0.001
**NYHA class**				0.293
I	76 (22%)	26 (27%)	50 (20%)	
II	150 (43%)	40 (42%)	110 (43%)	
III	124 (35%)	30 (31%)	94 (37%)	
IV	0 (0%)	0 (0%)	0 (0%)	
**History**				
Hypertension, n (%)	207 (59%)	57 (59%)	150 (59%)	0.957
Diabetes mellitus, n (%)	96 (27%)	22 (23%)	74 (29%)	0.245
Smoking, n (%)	161 (46%)	52 (54%)	109 (43%)	0.059
Ischemic cardiomyopathy, n (%)	178 (51%)	52 (54%)	126 (50%)	0.446
Atrial fibrillation, n (%)	61 (17%)	16 (17%)	45 (18%)	0.817
**Medications**				
Aspirin	247 (71%)	69 (72%)	178 (70%)	0.742
Digoxin	62 (18%)	19 (20%)	43 (17%)	0.531
β-Blocker, n (%)	327 (93%)	88 (92%)	239 (94%)	0.413
ACE inhibitor or ARB, n (%)	313 (89%)	85 (89%)	228 (90%)	0.740
Antiarrhythmic drugs, n (%)	22 (6%)	7 (7%)	15 (6%)	0.634
**CMR features**				
LV end-diastolic volume index, mL/m^2^	119 (96, 146)	129 (104, 155)	115 (94, 142)	0.026
LV end-systolic volume index, mL/m^2^	84 (65, 114)	97 (69, 125)	79 (65, 111)	0.049
LV ejection fraction, %	26 (20, 34)	26 (18, 32)	27 (20, 34)	0.183
LV LGE gray zone, g	5.3 (0.0, 15.7)	10.8 (1.8, 17.6)	4.6 (0.0, 14.5)	0.060
LV LGE core, g	9.2 (0.0, 21.3)	17.2 (2.5, 24.8)	6.7 (0.0, 18.9)	0.003
LV LGE total, g	16.0 (0.0, 38.9)	27.2 (5.4, 47.2)	13.7 (0.0, 35.9)	0.011
LA maximum volume index, mL/m^2^	41 (31, 58)	45 (33, 67)	40 (30, 57)	0.023
LA minimum volume index, mL/m^2^	24 (16, 41)	28 (19, 49)	23 (16, 39)	0.016
LA pre-atrial contraction volume index, mL/m^2^	36 (26, 51)	39 (28, 59)	33 (25, 49)	0.019
LA total emptying fraction, %	38 (26, 49)	35 (23, 47)	40 (28, 49)	0.022
LA passive emptying fraction, %	13 (7, 20)	13 (7, 19)	13 (7, 21)	0.847
LA active emptying fraction, %	27 (16, 37)	24 (12, 33)	29 (18, 39)	0.004

Data are median (quartile 1, quartile 3) or n (%). *NYHA* New York Heart Association, *ACE* angiotensin-converting enzyme, *ARB* angiotensin receptor blockers, *CMR* cardiac magnetic resonance, *LV* left ventricular, *LGE* late gadolinium enhancement, *LA* left atrial.

**Table 3:** Incidence rate and cine risk score for each endpoint.

	Incidence rate (100 person-years, 95% CI)	Event		No event		p value
		n (%)	Cine risk score	n (%)	Cine risk score	
Ventricular arrhythmia	4.57 (3.74–5.59)	96 (27)	0.60 (0.28, 0.82)	254 (73)	0.38 (0.14, 0.70)	< 0.001
Heart failure death	2.08 (1.58–2.72)	52 (15)	0.31 (0.11, 0.64)	298 (85)	0.13 (0.05, 0.37)	< 0.001
All-cause death	5.63 (4.77–6.64)	141 (40)	0.43 (0.19, 0.68)	209 (60)	0.23 (0.09, 0.47)	< 0.001

Cine risk score is shown as median (quartile 1, quartile 3). Cine risk score between different endpoints cannot directly be compared because the network was re-trained for each outcome separately.

### Cine risk score as a predictor

**Figure 1:**
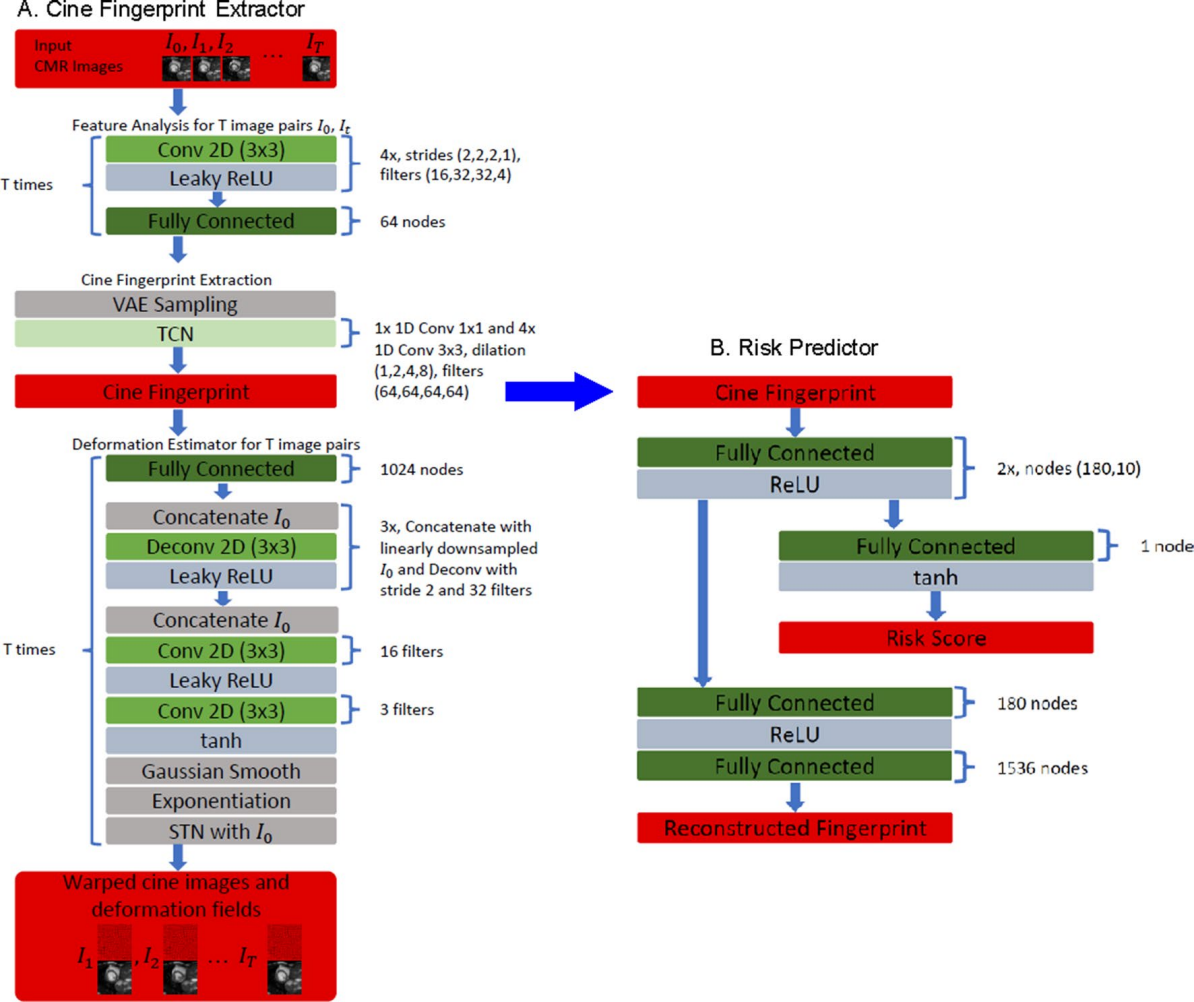
Algorithm overview. (**A**) Cine fingerprint extractor. (**B**) Risk predictor. See text for details.

**Figure 2:**
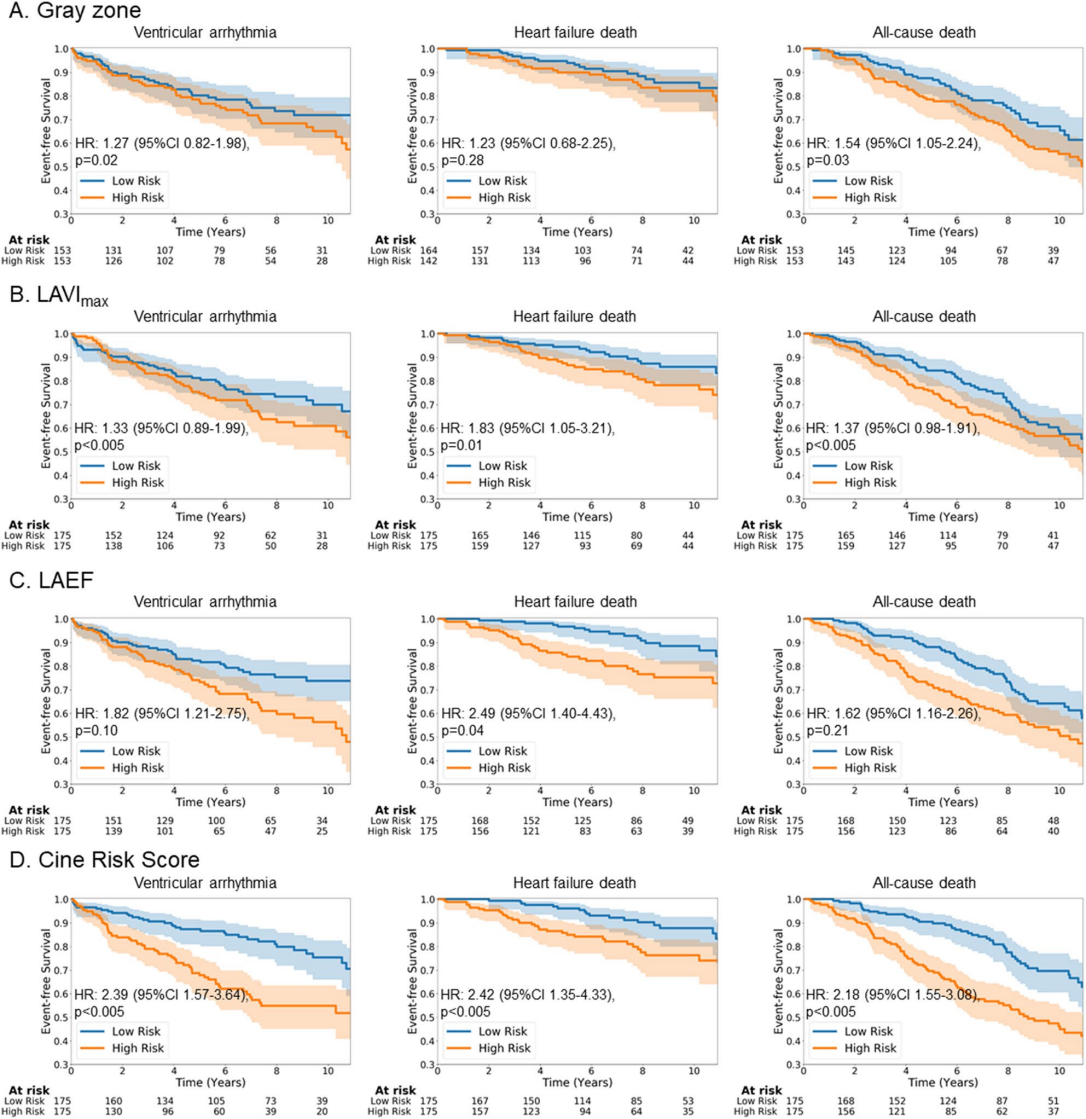
Survival prediction for each endpoint. (**A**) LV LGE gray zone. (**B**) LA maximum volume index, (**C**) LA total emptying fraction, (**D**) Cine risk score. The shaded area represents 95% confidence interval.

First, we assessed the value of cine fingerprint to predict outcomes within the training cohort by applying a univariate Cox hazards model to cine risk scores calculated by the risk predictor autoencoder network (Fig. 1). For all the endpoints, the cine risk score calculated from the cine fingerprint was significantly higher in those with compared to without events (Table 3). In addition, C-index of the cine risk score was higher than that of any other independent predictor including LV LGE gray zone, LA maximum volume index and LA total emptying fraction (Table 4). For VA and all-cause death, HR of the cine risk score was also higher than that of the other independent predictors. For HF death, the hazard ratio (HR) of cine risk score was lower than that of LA total emptying fraction. Survival analysis up to 10 years also showed that the cine risk score is a significant predictor for all the endpoints studied (Fig. 2). The cine risk score that is equal to or lower than the cut-off value of 0.15 (= 25 percentile) identifies a low-risk subgroup that achieved 83% VA-free survival at 10 years. This corresponds to an incidence rate of 2.55 per 100 person-years [95% CI 1.56–4.16], which is a 44% reduction from the incidence rate for the overall cohort of 4.57 per 100 person-years [95% CI 3.74–5.59], Table 3. Competing risk analysis showed that the cine risk score remained significantly associated with VA (subhazard ratio 3.82 [95% confidence interval 2.04–7.15], p < 0.001). Net reclassification improvement (NRI) index of cine risk score was 0.111 compared with LV LGE gray zone.

**Table 4:** Univariate Cox hazards model of cine risk scores calculated by the risk predictor autoencoder network.

Feature	Ventricular arrhythmia		Heart failure death		All-cause death	
	C-Index (95% CI)	HR (95% CI)	C-Index (95% CI)	HR (95% CI)	C-Index (95% CI)	HR (95% CI)
LV LGE gray zone	0.58 (0.52–0.62)	1.27 (0.82–1.98)	0.56 (0.46–0.65)	1.23 (0.68–2.25)	0.58 (0.53–0.63)	1.54 (1.05–2.24)
LA maximum volume index	0.56 (0.51–0.62)	1.33 (0.89–1.99)	0.63 (0.55–0.71)	1.83 (1.05–3.21)	0.60 (0.55–0.66)	1.37 (0.98–1.91)
LA total emptying fraction	0.58 (0.51–0.63)	1.82 (1.21–2.75)	0.68 (0.62–0.74)	2.49 (1.40–4.43)	0.62 (0.58–0.67)	1.62 (1.16–2.26)
Cine risk score	0.69 (0.64–0.75)	2.39 (1.57–3.64)	0.77 (0.69–0.85)	2.42 (1.35–4.33)	0.70 (0.66–0.78)	2.18 (1.55–3.08)
Gray zone + LAVI_max_ + LAEF	0.61 (0.55–0.66)	2.27 (1.42–3.60)	0.62 (0.53–0.69)	1.54 (0.84–2.84)	0.59 (0.53–0.66)	1.53 (1.06–2.21)
Cine risk score + Gray zone + LAVI_max_ + LAEF	0.67 (0.62–0.73)	2.37 (1.50–3.79)	0.77 (0.68–0.86)	2.61 (1.36–5.02)	0.68 (0.63–0.74)	1.75 (1.20–1.75)

*CI* confidence interval, *HR* hazard ratio, *LV* left ventricular, *LGE* late gadolinium enhancement, *LA* left atrial.

### Multivariate analysis

**Figure 3:**
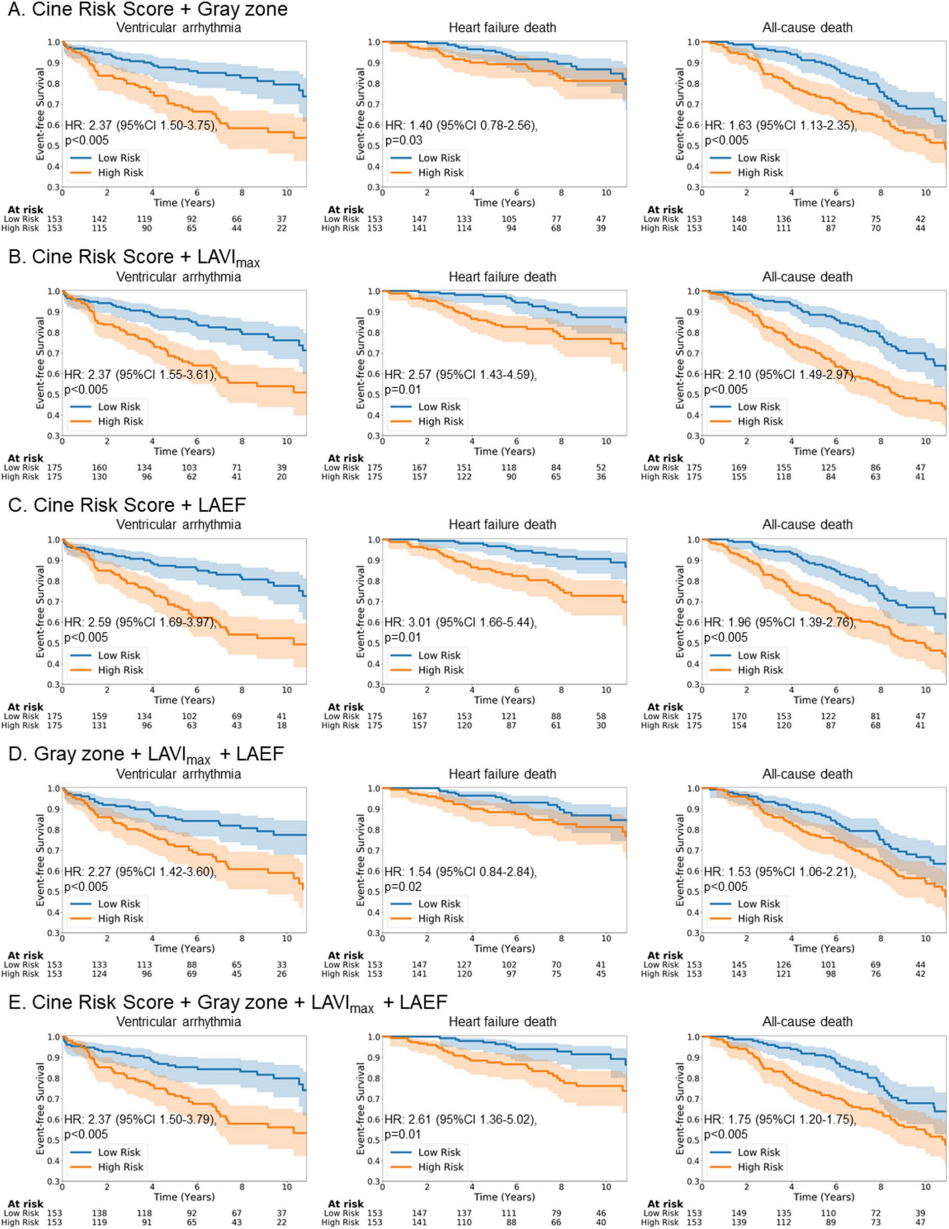
Survival prediction for each endpoint using multivariate Cox Proportional Hazards Regression models. (**A**) Cine risk score + LV LGE gray zone. (**B**) Cine risk score + LA maximum volume index. (**C**) Cine risk score + LA total emptying fraction. (**D**) LV LGE gray zone + LA maximum volume index + LA total emptying fraction. (**E**) Cine risk score + LV LGE gray zone + LA maximum volume index + LA total emptying fraction. The shaded area represents 95% confidence interval.

Next, we assessed the covariates together within multivariate Cox Proportional Hazards Regression models (Fig. 3). We performed survival analysis when combining the cine risk score with each of the predictors (LV LGE gray zone, LA maximum volume index and LA total emptying fraction) independently and performed a multivariate analysis with all covariates with and without the cine risk score. In each case, the addition of the cine risk score (e.g. Fig. 3A–C) improved the hazard ratio of each endpoint compared with the covariates without the cine risk score (e.g. Fig. 2A–C, respectively). In addition, the C-indices of the multivariate model with cine risk score also demonstrate the incremental value of the cine risk score (Table 4).

We also used unadjusted and adjusted Cox proportional regression analyses to assess the performance of cine risk score to predict the primary endpoint (Table 5). Univariate, unadjusted analysis identified male sex, use of diuretic as contributors of VA. After adjusting for sex, type of cardiomyopathy, use of diuretics, and hsCRP, cine risk score remained significantly associated with VA (HR 3.24, p = 0.005; Model 1). After further adjusting for LVEDI, LV ejection fraction, LV LGE gray zone, LA maximum volume index and LA total emptying fraction, cine risk score remained significantly associated with VA (HR 2.67, p = 0.027; Model 2 in Table 5). We applied the same analysis approach to the secondary endpoints. Cine risk score remained significantly associated with heart failure death after adjusting for age, NYHA class, duration and type of cardiomyopathy, history of diabetes, use of diuretics and digoxin (HR 5.62, p < 0.001; Model 1 in Supplementary Table 1). However, cine risk score was not significantly associated with heart failure death after additionally adjusting for LVEDI, LV ejection fraction, and LV LGE gray zone (HR 2.51, p = 0.119; Model 2 in Supplementary Table 1). In contrast, cine risk score remained significantly associated with all-cause death after adjusting for age, NYHA class, duration and type of cardiomyopathy, history of diabetes, use of diuretics, LVEDI, LV ejection fraction, and LV LGE gray zone (HR 2.27, p = 0.019; Model 2 in Supplementary Table 2).

**Table 5:** Predictors of ventricular arrhythmia by unadjusted and adjusted Cox proportional regression analysis.

Variables	Unadjusted		Model 1		Model 2	
	HR (95% CI)	p value	HR (95% CI)	p value	HR (95% CI)	p value
**Clinical features**						
Sex (female)	0.58 (0.34–0.96)	0.036	Included		Included	
Age, years	1.00 (0.99–1.02)	0.87	–		–	
Duration of CM, years	1.02 (0.99–1.06)	0.206	–		–	
Ischemic CM	1.09 (0.73–1.63)	0.68	Included		Included	
History of AF	0.94 (0.55–1.60)	0.81	–		–	
Use of diuretic	2.02 (1.29–3.15)	0.002	Included		Included	
hsCRP, mg/L	1.01 (0.99–1.02)	0.088	Included		Included	
**CMR features**						
LV EDVI, mL/m^2^	1.01 (1.00–1.01)	0.001	1.01 (1.00–1.01)	0.015	Included	
LV ESVI, mL/m^2^	1.01 (1.00–1.01)	0.003	1.00 (1.00–1.01)	0.034	–	
LV ejection fraction, %	0.98 (0.96–1.00)	0.057	0.99 (0.96–1.01)	0.190	Included	
LV LGE gray zone, g	1.02 (1.00–1.04)	0.029	1.03 (1.01–1.05)	0.009	Included	
LV LGE core, g	1.02 (1.01–1.03)	0.003	1.03 (1.01–1.05)	< 0.001	–	
LV LGE total, g	1.01 (1.00–1.02)	0.006	1.02 (1.01–1.03)	0.001	–	
LAVI_max_, mL/m^2^	1.02 (1.01–1.03)	0.001	1.01 (1.00–1.02)	0.032	Included	
LAVI_min_, mL/m^2^	1.02 (1.01–1.03)	< 0.001	1.01 (1.00–1.03)	0.014	–	
LAVI_preA_, mL/m^2^	1.02 (1.01–1.03)	< 0.001	1.01 (1.00–1.03)	0.026	–	
LA total emptying fraction, %	0.98 (0.96–0.99)	0.001	0.98 (0.96–0.99)	0.046	Included	
LA passive emptying fraction, %	0.99 (0.97–1.02)	0.57	1.00 (0.97–1.02)	0.961	–	
LA active emptying fraction, %	0.97 (0.96–0.99)	< 0.001	0.98 (0.96–0.99)	0.025	–	
Cine risk score	4.71 (2.45–9.08)	< 0.001	3.24 (1.43–7.38)	0.005	2.67 (1.12–6.37)	0.027

*Model 1* each CMR feature is adjusted for sex, type of CM, use of diuretics, and hsCRP in separate models. *Model 2* fully adjusted multivariable model incorporating Model 1, LVEDI, LV ejection fraction, LV LGE gray zone, LA maximum volume index and LA total emptying fraction. *HR* hazard ratio, *CI* confidence interval, *CM* cardiomyopathy, *AF* atrial fibrillation, *hsCRP* high-sensitivity C-reactive protein, *CMR* cardiac magnetic resonance, *LV* left ventricular, *EDVI* end-diastolic volume index, *ESVI* end-systolic volume index, *LGE* late gadolinium enhancement, *VI*_*min*_ minimum volume index, *VI*_*max*_ maximum volume index, *VI*_*preA*_ pre-atrial contraction volume index.

## Discussion

### Main findings

Our main findings are summarized as follows: (1) cine CMR inherently contains features of cardiac structure and function that improve VA risk prediction in primary prevention ICD candidates; (2) deep learning can automatically extract those features in the form of a cine risk score; and (3) the cine risk score is an independent biomarker of risk associated with VA and all-cause death in primary prevention ICD candidates. To our knowledge, this is the first study to demonstrate the incremental prognostic value of cardiac structure and function assessed from cine CMR in primary prevention ICD candidates. Earlier studies on predictive biomarkers for VA mainly focused on the presence, extent, and characteristics of myocardial scar detected by LGE CMR^16^. Its rationale is based on a electrophysiological assumption that the gray zone represents transitional tissues between scar and normal myocardium, and that slow conduction within the gray zone serves as a substrate for reentrant arrhythmia^17^. More recent studies identified LA function^10, 18, 19^, quantified by echocardiography or cine CMR, as independent predictors of SCD. However, quantification of chamber dysfunction in prior studies relied on pre-specified feature extraction with chamber segmentation in multiple imaging views. In contrast, our algorithm automatically extracts features from only cine CMR in an unsupervised fashion. In addition, our findings showed that the predictive value of cine risk score is independent of LA function (Supplementary Table 1), which suggests that cine CMR inherently contains predictive features beyond LA function.

### Mechanistic implications

Although deep learning is emerging as a powerful tool for diagnosis^11, 20^ and risk stratification^12^, it suffers from a lack of transparency and explainability^21^. In our study, the algorithm does not identify specific 4cv cine CMR function features associated with increased risk for VA. One possibility is imaging features associated with structural and functional interactions among the four chambers. Neural network-based algorithms can handle high-dimensional vector space simultaneously. This ability enables assessment of feature interactions as emergent phenomena that cannot be evaluated by studying each feature in isolation. Another possibility is imaging features associated with cardiac hemodynamics such as increased LV filling pressures, decreased LV compliance, increased LV wall stress associated with clinical heart failure. Because low RV^9, 22^ and LA function^10, 18, 19^ are independent predictors of SCD, it is possible that the algorithm identified imaging features of impending biventricular failure associated with VA. Importantly, VA risk prediction approaches to date have not comprehensively incorporated metrics from these two possibilities, which are not mutually exclusive. Future studies are needed to address the knowledge gap as to the potential clinical significance of these metrics in VA prediction.

### Clinical implications

The proposed algorithm has a potential clinical impact to help primary prevention ICD candidates and their physicians make an informed decision regarding ICD implantation during the shared decision making process^23^. Notably, the algorithm is applied to 4cv cine CMR, which does not require intravenous contrast agents. This is particularly important for individuals with severe HF and cardiorenal syndrome who are considered for primary prevention ICD. The key innovation of the developed algorithm is that it is does not require manual segmentation of the heart chambers, which allows quicker risk assessment without cognitively biased human intervention.

### Solicitation of participation to contribute to external validation cohort

Our findings clearly indicate that non-contrast, cine CMR inherently contains features that improve VA risk prediction in primary prevention ICD candidates. However, the training cohort is of relatively small sample size and derives from a single institution. To assess the generalizability of the algorithm, it needs to be tested in an external validation cohort. The unique value of the CERTAINTY study is the long follow-up duration (10 years), because, unlike pharmacologic or ablation interventions, ICD is usually a lifetime commitment. The ICD candidates need to be informed of long-term implications at the time of shared decision making on ICD implantation. This unique value of long-term follow-up unfortunately limits the data availability of the CERTAINTY study. Even with the advent of remote ICD monitoring, most institutions have very few data sets with this duration of follow-up. Therefore, we encourage participation of multiple institutions in the CERTAINTY study by contributing to the external validation cohort. The baseline characteristics of the CERTAINTY population is described in Table 1. The external validation cohort should meet two important criteria. First, the baseline characteristics of the external validation cohort should be clinically matched to those of the training cohort. Critically important variables to be matched include the follow-up duration and the etiology of cardiomyopathy (ischemic vs. nonischemic). The follow-up duration is of particular importance to accrue an adequate number of events to improve rigor. Second, the external validation cohort should have a sufficiently large sample size to draw statistically meaningful conclusions. We estimate the sample size based on the event-free survival of heart failure death in the training cohort, because the incidence rate of heart failure death was the lowest among the three main outcomes (ventricular arrhythmia, heart failure death and all-cause death) (Table 3). The expected freedom from heart failure death at 10 years in the low- and high-risk group based on the cine risk score was 0.877 (95% CI 0.801–0.925) and 0.740 (95% CI 0.642–0.815), respectively (Fig. 2D). Based on those values, we determined that 344 cases are needed for the external validation cohort to have a power of 90% at two-sided alpha level of 0.05.

### Limitations

There are two limitations associated with the study. First, we used only 4cv cine CMR, which was included in a routine image acquisition protocol. Therefore, it is possible that the algorithm underestimated the degree of abnormal structure and function by missing regions that were not covered by the 4cv view. However, we believe that the advantage of our approach outweighs the disadvantage of including multiple views to assess 3-D structure and function, which would increase the scan time and post-processing burden including a higher training complexity for the algorithm. Second, the proposed method focuses on the extraction of cardiac function features while additional imaging features capturing anatomic abnormalities could further improve the risk prediction.

## Conclusions

Non-contrast, cine CMR inherently contains imaging features that can improve VA risk prediction in primary prevention ICD candidates without the need for manual contouring or contrast-enhancement. The deep learning algorithm could be easily implemented in routine clinical practice and provide valuable information during the shared decision-making process.

## Methods

### Training cohort

The protocol was approved by the institutional review board of the Johns Hopkins Medical Institutions, and all the patients provided written informed consent. All methods were carried out in accordance with relevant guidelines and regulations. We retrospectively analyzed a training cohort with ICM and NICM who underwent CMR at Johns Hopkins Medical Institutions (Baltimore, MD) using 1.5-Tesla whole body scanners prior to primary prevention ICD implantation (median 3 days) between 2003 and 2015 with a standard imaging protocol (*Left Ventricular Structural Predictors of SCD*, ClinicalTrials.gov Identifier: NCT01076660). The device type included a single-, dual-chamber ICD, and CRT-D based on current guidelines^1^. Patients were evaluated every 6 months and after any ICD shock. Patients who were not seen in-person underwent a telephone interview to update history. Out of this cohort, we have previously reported the association between LGE gray zone extent and VA inducibility at electrophysiology study (n = 47 with ICM)^24^, or appropriate ICD firing (n = 235 with ICM and NICM)^8^, the association between LGE-based arrhythmia simulation and appropriate ICD firing (n = 41 with ICM)^25^, the association between LGE scar characteristics and LVEF improvement (n = 202 with ICM and NICM)^26^, the association between LA function and inappropriate ICD firing (n = 162 with ICM and NICM)^27^, the association between LGE scar complexity and VA (n = 122 with ICM)^28^, and the association between time-varying risk covariates and appropriate ICD firing (n = 382 with ICM and NICM)^29^. In this study, 350 patients out of this cohort were included where both LGE and 4cv cine CMR images were available to test whether a deep learning algorithm can extract features associated with VA from 4cv cine CMR images in an unsupervised fashion. The primary outcome was defined as adjudicated appropriate ICD shock or appropriate anti-tachycardia pacing (ATP) for VA, including ventricular tachycardia and fibrillation. The secondary endpoint was death due to HF and all-cause death. Deaths were classified according to the most proximate cause after review of ICD interrogations, medical records, death certificates, autopsy reports, and eyewitness accounts.

### CMR imaging and analysis

The cohort (n = 350) were studies in one of two different types of scanners (MAGNETOM Avanto, 1.5 Tesla, Siemens Healthcare, Erlangen, Germany [n = 263, 75%] and Signa CV/I, 1.5 Tesla, GE Healthcare, Milwaukee, WI [n = 87, 25%]). Details of CMR imaging and analysis are described in Supplementary Appendix 1 and Supplementary Fig. 1. Briefly, short- and long-axis cine images were acquired with a steady-state free precession sequence. Two- (2-D) or three-dimensional (3-D) LGE cross-sectional short- and long-axis images were acquired starting at approximately 15 min after intravenous administration of 0.15–0.20 mmol/kg of gadolinium-based contrast agent. Typical parameters were TR = 5.4–8.3; TE = 1.3–3.9; TI optimized for nulling of normal myocardium; spatial resolution 1.4–1.5 × 2.2–2.4 × 8 mm. Two observers analyzed all LGE images acquired from both scanners using research software (Cinetool, GE Healthcare, Milwaukee, WI). The core scar and the gray zone were quantified as all pixels with signal intensity (SI) > 50% of maximal SI within the hyper-enhanced region, and SI greater than the peak SI in the normal myocardium but < 50% of the maximal, respectively^8^. Multimodality Tissue Tracking software (MTT, version 6.0, Canon Medical Systems, Japan) was used to obtain phasic LA volumes, strain, and strain rate from four-chamber cine CMR images^27^.

### Algorithm development

In this work, the above-mentioned cine CMR dataset was used to train two independent neural networks. First, a probabilistic encoder-decoder neural network^14^ was trained to extract cardiac structure and function features from 4cv cine CMR in a form of cine fingerprint in a fully unsupervised fashion (*Cine Fingerprint Extractor*, Fig. 1A). Each of the 4cv cine CMR images was cropped to include only the heart within a square of 128 × 128 pixels with a spacing of 2.2 mm. An L2 reconstruction error term, a regularizer acting on the distributions of the latent space, was used to learn a probabilistic fingerprint space. The network was trained using sixfold cross-validation (using 5/6 of the cases for training and 1/6 for validation for each fold). Second, an autoencoder neural network was trained by regressing disease outcomes as a cine risk score (0 to 1 probability scale) with the cine fingerprint as an input (*Risk Predictor,* Fig. 1B). The network was trained using sixfold cross-validation and was re-trained for each outcome separately (VA, heart failure death, and all-cause death). To enable the use of censored data, a partial likelihood loss function derived from Cox’ semi-parametric proportional hazards model was utilized^30^. To ensure data cannot leak between both networks, the training procedure was repeated 6 times by selecting a different fold as the validation data set. The results of all 6 validation groups from the different trainings were then combined to evaluate the complete dataset. In addition the Risk Predictor network was evaluated using bootstrapping^31^ for measuring the performance. Further details of the algorithm development are described in Supplementary Appendix 2 and Supplementary Fig. 2. We implemented both neural networks using Keras (ver. 2.3, https://keras.io/, Google, LLC, Mountain View, CA, USA) with Tensorflow (ver. 1.14, https://www.tensorflow.org/, Google, LLC, Mountain View, CA, USA) backend.

### Statistical analysis

We used Pearson's *χ*^2^ test for categorical variables and the Student t-test or Mann–Whitney *U* test for parametric or nonparametric continuous variables, respectively. For risk prediction, we report the mean concordance (C-) index^32^ between predicted risk score and actual event time. We applied bootstrapping^31^ with resampling of 100 times for computing the C-index and CI to confirm the cross-validated results. We computed the hazard ratio (HR) including CI and p-value by fitting a linear Cox regression model on the predicted risk scores. To this end, the median risk value was used to divide the cohort in a low- and a high-risk group. Kaplan–Meier estimates for cumulative survival rates for the low- and high-risk groups were determined and statistically evaluated with the log-rank test. Cox proportional hazards models were used to estimate the association between variables and endpoints. Univariable analyses of all baseline variables were performed. Multivariable analyses were performed separately for each CMR feature by adjusting for clinical variables significantly associated with outcomes in univariable analysis (p < 0.05) and/or clinically deemed important (Model 1). We also performed competing risk analysis for the cumulative incidence of VA with death as a competing event, using the method by Fine and Gray^33^ and net reclassification improvement (NRI) analysis^34^. We used STATA (ver 16, Stata Corp LP, College Station, TX) and lifelines (ver 0.25, https://lifelines.readthedocs.io/) for statistical analysis. A two-sided p-value of < 0.05 was considered statistically significant.

## Supplementary Information

Supplementary Information.
