# Supplementary material for: CinE caRdiac magneTic resonAnce to predIct veNTricular arrhYthmia (CERTAINTY)
Source: Sci Rep. 2021 Nov 22;11:22683. doi: 10.1038/s41598-021-02111-7 (PMC8608832; doi:10.1038/s41598-021-02111-7)

SUPPLEMENTARY INFORMATION for

**CinE caRdiac magneTic resonAnce**

**to predIct veNTricular arrhYthmia (CERTAINTY)**

Julian Krebs, Tommaso Mansi, Hervé Delingette, Bin Lou, Joao A.C. Lima, Susumu Tao,

Luisa A. Ciuffo, Sanaz Norgard, Barbara Butcher, Wei H. Lee, Ela Chameta,

Timm-Michael Dickfeld, Michael Stillabower, Joseph E. Marine,

Robert G. Weiss, Gordon F. Tomaselli, Henry Halperin,

Katherine C. Wu, Hiroshi Ashikaga*

*To whom correspondence should be addressed. Email: [hashika1@jhmi.edu](mailto:hashika1@jhmi.edu)

**Supplementary Appendix 1: Cardiac magnetic resonance (CMR) imaging and analysis**

*CMR imaging.* Short- and long-axis cine images were acquired in breath hold with a steady-state free precession sequence (TR=2.5-3.8; TE=1.1-1.6; flip angle=40-60°; temporal resolution 25-45 msec; typical spatial resolution 1.5x2.4x8 mm). Two- (2-D) or three-dimensional (3-D) late gadolinium enhancement (LGE) cross-sectional short- and long-axis images through the left ventricle were acquired starting at approximately 15 minutes after intravenous administration of 0.15-0.20 mmol/kg of gadodiamide (Omniscan, GE Healthcare) or gadopentetate dimeglumine (Magnevist, Schering AG) using an inversion-recovery fast gradient-echo sequence (TR=5.4-8.3; TE=1.3-3.9; TI optimized for nulling of normal myocardium; typical spatial resolution 1.4-1.5x2.2-2.4x8 mm).

*CMR analysis – LGE.* Two observers analyzed LGE images using research software (Cinetool, GE Healthcare, Milwaukee, WI, and Segment v2.0, Medviso AB, Lund, Sweden) The core scar and the gray zone were quantified as all pixels with signal intensity (SI) >50% of maximal SI within the hyper-enhanced region, and SI greater than the peak SI in the normal myocardium but <50% of the maximal, respectively.

*CMR analysis – Left atrial (LA) structure and function.* Multimodality Tissue Tracking software (MTT, version 6.0, Canon Medical Systems, Japan) was used to obtain phasic LA volumes, strain, and strain rate from four-chamber cine CMR images (**Supplementary Figure** **1**). Details of the MTT and its validation and reproducibility have been described previously. LA endocardial and epicardial borders were manually traced in the 4-chamber view (4cv) image at the end of LV systole, excluding pulmonary veins and LA appendage. The software automatically tracks on screen pixels during the cardiac cycle. Maximum, minimum, and pre-atrial contraction LA volume (V_max_, at end systole before mitral valve opening; V_min_, at end diastole immediately after mitral valve closure; V_preA_, before atrial contraction, respectively) were measured using the LA volume curve generated by the Simpson’s method. All measured LA volumes were subsequently indexed according to body surface area. The LA volumetric indices were calculated as follows: LA total emptying fraction = (V_max_ - V_min_) ×100% / V_max_; LA passive emptying fraction = (V_max_ - V_preA_) ×100% / V_max_; and LA active emptying fraction = (V_preA_ - V_min_) ×100% / V_preA_. All CMR analyses were performed blinded to clinical outcomes.

**Supplementary Appendix 2: AI algorithm**

*Cine Fingerprint Extractor*. The cardiac cine fingerprint contains automatically derived features of the cardiac function from 4cv cine CMR. These features are learned as latent variables $z$ in an encoder-decoder neural network for image sequences. In a first step, the encoder extracts a pair-wise fingerprint $\tilde{z}_{t}\in\mathbb{R}^{D}$ that depicts $D$ function features between two frames of the image sequence, the first frame $I_{0}$ and the frame $I_{t}$ at time step $t$. This intermediate fingerprint $\tilde{z}_{t}$ is extracted in parallel for all image pairs $(I_{0}, I_{t})$ from image sequences of one heartbeat with length $T$ where $t\in[1,T]$. The first frame $I_{0}$ is chosen to be the end-diastole frame as a convention.

In order to build a cine fingerprint that is consistent throughout the entire heartbeat, the sequence of encoded intermediate function features $\tilde{z}_{t}$ are combined using a temporal convolutional neural network (TCN) making each time step dependent on all past and future time steps. The output is the cine fingerprint $z\in\mathbb{R}^{D\times(T-1)}$ which represents a compact cardiac function encoding of the 4cv cine CMR with $D*(T-1)$ features. To ensure that the cine fingerprint contains cardiac function features, the decoder of the neural network uses the fingerprint $z_{t}$ at each time step $t$ to reconstruct the deformation field $\phi_{t}$ depicting the pixel-wise point correspondences between images $I_{0}$ and $I_{t}$. An overview of the architecture of the encoder-decoder neural network is shown in **Supplementary Figure 2A**.

The framework forms a latent variable model and is trained using a conditional variational autoencoder (CVAE). The encoder $q_{\omega}$ with network weights $\omega$ approximates the posterior distribution $q_{\omega}(\tilde{z}|I_{0:T})$. Second, as the key component of temporal modeling, the intermediate latent vectors $\tilde{z}_{t}$ are mapped to the cine fingerprint $z$ by combining all latent vectors of all time steps and condition them on the normalized time $\bar{t}$ using a TCN $p_{\gamma}$ with weights $\gamma$ (**Supplementary Figure 2B**): $p_{\gamma}(z|\tilde{z}_{1:T}, \bar{t}_{1:T})$. Finally, the decoder $p_{\theta}$ with trainable network weights $\theta$ aims to reconstruct the fixed image $I_{t}$ by warping the moving image $I_{0}$ using the predicted deformation field $\phi_{t}$. The decoder is conditioned on the temporally regularized $z_{t}$-codes and the image $I_{0}$ which is down-sampled and concatenated with the features at each decoder scale. The decoder corresponds to the data likelihood $p_{\theta}(I_{1:T}|z,I_{0})$.

During training, a lower bound on the data likelihood is maximized with respect to a prior distribution $p(\tilde{z}_{t})$ in order to retrieve a structured latent space $\tilde{z}_{t}$ (cf. CVAE). The prior ${p(\tilde{z}}_{t})$ is assumed to follow a multivariate unit Gaussian distribution with spherical covariance $I$: ${p(\tilde{z}}_{t})\mathcal{\sim N}(0,I)$. The loss function of the fingerprint extractor results in optimizing the expected log-likelihood $p_{\theta}$ and the Kullback-Leibler (KL) divergence enforcing the posterior distribution $q_{\omega}$ to be close to the prior ${p(\tilde{z}}_{t})$ for all time steps:

$$\mathcal{L}_{\mathrm{fingerprint}}\left( \omega,\gamma,\theta\right)=\sum_{t=1}^{T} -\mathbb{E}_{z_{t}\sim p_{\gamma}\left( \cdot|\tilde{z}_{1:T}, \bar{t}_{t:T} \right)}[\log p_{\theta}\left( I_{t}|z_{t},I_{0} \right)]+\mathrm{KL} [q_{\omega}\left( \tilde{z}_{t}|I_{0},I_{t} \right)\left| p\left( \tilde{z}_{t} \right) \right].$$

Unlike the traditional CVAE model, the temporal regularized $z_{t}$-code is used in the log-likelihood term $p_{\theta}$ instead of the $\tilde{z}_{t}$. We model $p_{\theta}$ as a symmetric local cross-correlation Boltzmann distribution with the weighting factor $\iota$. All network weights except the ones in the TCN are shared and thus independent of the time $t$. Their network architecture consists of convolutional and deconvolutional layers with fully-connected layers for mean and variance predictions in the encoder part. We use an exponentiation layer for a stationary velocity field parameterization of diffeomorphisms and the output was smoothed using a Gaussian kernel to ensure folding-free deformations. Furthermore, a linear warping layer (spatial transformer network) was applied using linear interpolation was used to receive the deformed image sequence. During training, we apply temporal dropout sampling in order to further ensure learning temporal dependencies and increase generalizability.

We increased the latent dimensionality $D$ to 64 based on the fact that 4 chamber view images contain more complex cardiac function details than the left-ventricular function alone. The survival predictor requires cine fingerprints $z$ to have the same size for all patients. In order to retrieve same sized $z$, we interpolated the cine CMR in temporal dimension to retrieve a fixed time length $T$. In this work, we used $T=25$ as it represents the average sequence length in this cohort. We applied B-spline interpolation for resampling the image sequences that contained more or less than 25 frames. The Gaussian deformation field regularization was applied with a smoothing of $3$mm in spatial and $1.5$mm in termporal dimension. The weighting factor between reconstruction and KL loss terms has been chosen empirically as $\iota= 6\cdot{10}^{-4}$. The dropout sampling probability was 0.5.

The neural network (**Figure 1A**) contained 4 convolutional layers with Leaky ReLU activation function in the encoder with (16, 32, 32, 4) filters and strides of (2, 2, 2, 1). The latent space contained a sampling operation according to CVAEs and a TCN with one one-dimensional (1-D) 1x1 convolution and 4 1-D convolutions with dilations (1, 2, 4, 8), ReLU activation functions and skip connections. The decoder, contained 3 blocks of concatenations and deconvolutional layers with Leaky ReLU activation functions, 32 filters and a stride of 2. In each block, the input has been concatenated with a linearly downsampled version of the end-diastolic frame. After the deconvolutional blocks, 2 convolutional layers with Leaky ReLU, *tanh* activation functions and (16, 3) filters were applied. The loss function was two-fold, containing a data fidelity term (local cross-correlation between warped and target image) and a probabilistic regularizer enforcing all variable of the fingerprint to follow a unit Gaussian distribution. We applied a first-order gradient-based method for stochastic optimization (Adam) with a learning rate of 0.00015 and a batch size of one. During training, we performed data augmentation on-the-fly by randomly shifting, rotating, scaling and mirroring image sequences.

*Risk Predictor*. The risk predictor neural network takes the cine fingerprint $z$, the compact representation of the cardiac structure and function, as input and predicts the survival risk score $r$. As in standard Cox regression analysis, the risk score is a scalar number that is constant in time. It is larger for a subject with a higher probability of experiencing an event and is defined by the logarithm of the hazard ratio. This ratio contains the hazard $h_{z}(t)$ of a subject with fingerprint $z$ with respect to the baseline hazard $h_{0}(t)$:

$$r = \log\frac{h_{z}(t)}{h_{0}(t)}.$$

The hazard $h_{z}(t)$ symbolizes the probability of the subject of dying at time $t$ and the baseline hazard describes the survival without an influence of features (or covariates) $z$. The hazard ratio is assumed to be constant over time behind the semi-parametric proportional hazard model of Cox. Thus, the continuous risk score $r$ allows to classify the outcome risk (e.g. into low and high risk groups) for a new patient at test time.

The loss function of the risk predictor was two-fold with one reconstruction loss term minimizing the distance between input and output of the encoder-decoder network. The second loss term used the negative log partial likelihood as survival function following standard Cox regression analysis.

In contrast to standard Cox regression analysis, we define the risk $r$ as a non-linear combination of input features $z$: $r=r_{\nu}(e_{\kappa}(z))$ where $r_{\nu}$ and $e_{\kappa}$ are two neural networks with network weights $\nu$ and $\kappa$. The full risk model is realized as autoencoder neural networks that reduce the fingerprint's dimensionality $D\bar{T}$in order to retrieve the risk $r$. The authors chose an encoder-decoder architecture in contrast to a direct prediction of $r$ in order to constrain and regularize the risk predictor to avoid over-fitting.

The encoding and decoding branches of the risk autoencoder are denoted by $e_{\kappa}$ and $d_{\lambda}$ with network weights $\kappa$ and $\lambda$ respectively. A third network with weights $\nu$ is applied to obtain the risk score $r_{\nu}(e_{\kappa}(z))$ from the latent space of the autoencoder $e_{\kappa}(z)$. The three networks consist of fully-connected layers due to the low-dimensional fingerprints. In case of larger fingerprints, convolutional and deconvolutional layers in encoder respectively decoder networks could be used. The risk predictor is trained using multi-task learning by aiming to reconstruct the cine fingerprint and to predict the risk $r$ at the same time. Thus, the loss function $\mathcal{L}_{\mathrm{risk}}(\kappa, \lambda, \nu)$ contains 2 terms, one for the fingerprint reconstruction $\mathcal{L}_{\mathrm{rec}}(\kappa,\lambda)$ and one for risk prediction $\mathcal{L}_{\mathrm{risk}}(\kappa,\nu)$:

$$\mathcal{L}_{\mathrm{risk}}\left( \kappa, \lambda, \nu\right)= \mathcal{L}_{\mathrm{rec}}\left( \kappa,\lambda\right)+\alpha\mathcal{L}_{\mathrm{risk}}\left( \kappa,\nu\right),$$

where $\alpha$ denotes a weighting factor between both terms. For risk prediction, we apply the negative log partial likelihood as survival function over $N$ censored training samples following standard Cox regression analysis:

$$\mathcal{L}_{\mathrm{risk}}\left( \kappa,\nu\right)= -\sum_{i=1}^{N} \delta_{i}\left[ r_{\nu}\left( e_{\kappa}\left( z^{i} \right) \right)-\log\sum_{j=1}^{N} R_{ij} exp\left( r_{\nu}\left( e_{\kappa}\left( z^{j} \right) \right) \right) \right],$$

with $z^{i}$ being the fingerprint of the $i$-th training subject. The Boolean censoring indicator $\delta_{i}$ equals $1$ if the subject experienced an endpoint at the given time $\tau$. A subject is censored $\delta_{i}=0$ if the patient was still alive at time $\tau$ but removed from the study afterwards. $R$ is the risk matrix where $R_{ij}=1$ if $\tau_{j}\geq\tau_{i}$ and $R_{ij}=0$ if $\tau_{j} <\tau_{i}$, based on $N$ training samples per batch. This represents a non-linear Cox proportional hazard model. The fingerprint reconstruction loss term is defined as the mean squared error between fingerprint $z$ and reconstructed fingerprint $z'=d_{\lambda}(e_{\kappa}(z))$:

$$\mathcal{L}_{\mathrm{rec}}\left( \kappa,\lambda\right)= \frac{1}{N}\sum_{i=1}^{N} |z^{i}-d_{\lambda}(e_{\kappa}\left( z^{i} \right)|^{2}.$$

The architecture of the Risk Predictor contained 5 fully-connected layers (**Figure 1B**). A dropout factor of 0.3 has been applied on the input layer of the risk predictor. First, 2 fully-connected layers with ReLU activations were applied that reduced the cine fingerprint dimensionality to 180 and 10 subsequently. Second, the resulting vector was input to another fully-connected layer with *tanh* activation function that outputs a 1-D risk score. For training stability, a decoder that reconstructs the cine fingerprint from the encoded ten-dimensional (10-D) feature vector was also established. This decoder contained 2 fully-connected layers with ReLU and linear activation function. The weighting factor of the two loss terms has been set to 0.5. The Risk Predictor was trained using the Adam optimizer with a learning rate of 0.0001 and batch size of 16. Dropout factor and number of units of the fully-connected layers were determined by a hyperparameter search using evolutionary optimization on the first training fold.

The two networks were trained in two steps. First, the cine fingerprint was trained alone and afterwards the survival predictor while fixing the cine fingerprint network. This kept the fingerprint independent of the survival analysis and allowed for example the training on additional data for fingerprint extraction where no survival data is available. We applied stratified 6-fold cross-validation training schemes for both networks, keeping similar relative event and non-event occurrences in all folds. Keeping the 6 validation folds allows to obtain validation results of all the data set and guaranteeing no data leakage.

**Supplementary Figure Legends**

**Supplementary Figure 1. Left atrial (LA) structure and function analysis using tissue-tracking cardiac magnetic resonance (CMR).** *A. Planimetry of the LA endocardial and epicardial borders were manually performed in the 4-chamber view (4cv) cine CMR view at the end of left ventricular (LV) systole.* *B. LA wall motion was tracked across a cardiac cycle (shown at LV end-diastole****)***. *C. LA maximum volume (V_max_), minimum volume (V_min_), and pre-atrial contraction volume (V_preA_) were identified from the LA volume curve, and LA total, passive and active emptying fraction (EF) were calculated.* RA, right atrium; RV, right ventricle.

**Supplementary Figure 2. Neural network architecture of the cine fingerprint extractor.** *(****A****) The encoder* $q_{\omega}$ *projects the image pair* $(I_{0}, I_{t})$ *to the intermediate fingerprint* $\tilde{z}_{t}$ *containing cardiac structure and function features. The temporal convolutional network* $p_{\gamma}$ *(****B****) constructs the cine fingerprint* $z$ *using the* $\tilde{z}_{t}$ *from all time steps, conditioned on the normalized time* $\bar{t}$*. The cine fingerprint* $z$ *can be understood as a low-dimensional cardiac function encoding which the decoder* $p_{\theta}$ *maps to the deformation fields* $\phi_{t}$ *while being conditioned on the image* $I_{0}$*.*

**Supplementary Table 1. Predictors of heart failure death by unadjusted and adjusted Cox proportional regression analysis.** *Model 1:* each CMR feature is adjusted for age, NYHA class, duration and type of CM, history of diabetes, use of diuretics, and use of digoxin in separate models. *Model 2:* fully adjusted multivariable model incorporating Model 1, LVEDI, LV ejection fraction, and LV LGE gray zone. Abbreviations as in Table 4.

| Variables | Unadjusted | | Model 1 | | Model 2 | |
| --- | --- | --- | --- | --- | --- | --- |
|  | HR (95%CI) | P value | HR (95%CI) | P value | HR (95%CI) | P value |
| **Clinical features** |  |  |  |  |  |  |
| Sex (female) | 0.94 (0.50-1.78) | 0.86 | - |  | - |  |
| Age, years | 1.03 (1.01-1.06) | 0.002 | Included |  | Included |  |
| NYHA class | 1.53 (1.05-2.25) | 0.027 | Included |  | Included |  |
| Duration of CM, years | 1.06 (1.02-1.10) | 0.004 | Included |  | Included |  |
| Ischemic CM | 0.89 (0.52-1.54) | 0.69 | Included |  | Included |  |
| History of AF | 1.38 (0.73-2.66) | 0.32 | - |  | - |  |
| Diabetes | 2.30 (1.31-4.02) | 0.004 | Included |  | Included |  |
| Use of diuretic | 3.28 (1.64-6.54) | 0.001 | Included |  | Included |  |
| Use of digoxin | 2.13 (1.19-3.81) | 0.011 | Included |  | Included |  |
| Serum hematocrit, % | 0.94 (0.88-0.99) | 0.040 | - |  | - |  |
| Serum BUN, mg/dL | 1.06 (1.03-1.08) | <0.001 | - |  | - |  |
| NT-proBNP | 1.00 (0.99-1.00) | 0.30 | - |  | - |  |
| hsCRP, mg/L | 1.02 (1.00-1.03) | 0.046 | - |  | - |  |
| **CMR features** |  |  |  |  |  |  |
| LV EDVI, mL/m^2^ | 1.01 (1.01-1.02) | <0.001 | 1.02 (1.01-1.02) | <0.001 | Included |  |
| LV ESVI, mL/m^2^ | 1.01 (1.01-1.02) | <0.001 | 1.02 (1.01-1.02) | <0.001 | - |  |
| LV ejection fraction, % | 0.93 (0.90-0.96) | <0.001 | 0.93 (0.90-0.96) | <0.001 | Included |  |
| LV LGE gray zone, g | 1.03 (1.00-1.05) | 0.028 | 1.04 (1.01-1.07) | 0.005 | Included |  |
| LV LGE core, g | 1.01 (0.99-1.03) | 0.16 | 1.02 (1.00-1.04) | 0.042 | - |  |
| LV LGE total, g | 1.01 (0.99-1.02) | 0.058 | 1.01 (1.00-1.03) | 0.011 | - |  |
| LAVI_max_, mL/m^2^ | 1.03 (1.02-1.04) | <0.001 | 0.99 (0.98-1.01) | 0.67 | - |  |
| LAVI_min_, mL/m^2^ | 1.03 (1.02-1.04) | <0.001 | 1.00 (0.98-1.01) | 0.89 | - |  |
| LAVI_preA_, mL/m^2^ | 1.03 (1.02-1.04) | <0.001 | 0.99 (0.98-1.02) | 0.94 | - |  |
| LA total emptying fraction, % | 0.96 (0.94-0.98) | <0.001 | 0.99 (0.96-1.02) | 0.43 | - |  |
| LA passive emptying fraction, % | 0.96 (0.93-0.99) | 0.01 | 0.98 (0.94-1.03) | 0.47 | - |  |
| LA active emptying fraction, % | 0.97 (0.95-0.99) | 0.007 | 0.99 (0.97-1.02) | 0.64 | - |  |
| Cine risk score | 8.35 (3.56-19.6) | <0.001 | 5.62 (2.23-14.2) | <0.001 | 2.51 (0.79-8.00) | 0.119 |

**Supplementary Table 2. Predictors of all-cause death by unadjusted and adjusted Cox proportional regression analysis.** *Model 1:* each CMR feature is adjusted for age, NYHA class, duration of CM, type of CM, history of diabetes, and use of diuretics. *Model 2*: fully adjusted multivariable model incorporating Model 1, LVEDI, LV ejection fraction, and LV LGE gray zone. Abbreviations as in Table 4.

| Variables | Unadjusted | | Model 1 | | Model 2 | |
| --- | --- | --- | --- | --- | --- | --- |
|  | HR (95%CI) | P value | HR (95%CI) | P value | HR (95%CI) | P value |
| **Clinical features** |  |  |  |  |  |  |
| Sex (female) | 0.95 (0.64-1.40) | 0.78 | - |  | - |  |
| Age, years | 1.04 (1.03-1.06) | <0.001 | Included |  | - |  |
| NYHA class | 1.47 (1.17-1.85) | 0.001 | Included |  | - |  |
| Duration of CM, years | 1.03 (1.00-1.06) | 0.037 | Included |  | - |  |
| Ischemic CM | 1.54 (1.09-2.20) | 0.014 | Included |  | - |  |
| History of AF | 1.16 (0.77-1.75) | 0.46 | - |  | - |  |
| Diabetes | 1.87 (1.32-2.66) | <0.001 | Included |  | - |  |
| Use of diuretic | 1.92 (1.33-2.77) | <0.001 | Included |  | - |  |
| Use of digoxin | 1.27 (0.85-1.88) | 0.24 | - |  | - |  |
| Serum hematocrit, % | 0.96 (0.92-0.99) | 0.022 | - |  | - |  |
| Serum BUN, mg/dL | 1.05 (1.03-1.06) | <0.001 | - |  | - |  |
| NT-proBNP | 1.00 (0.99-1.00) | 0.32 | - |  | - |  |
| hsCRP, mg/L | 1.01 (1.00-1.02) | 0.021 | - |  | - |  |
| **CMR features** |  |  |  |  |  |  |
| LV EDVI, mL/m^2^ | 1.01 (1.00-1.01) | <0.001 | 1.01 (1.01-1.01) | <0.001 | Included |  |
| LV ESVI, mL/m^2^ | 1.01 (1.00-1.01) | <0.001 | 1.01 (1.01-1.01) | <0.001 | - |  |
| LV ejection fraction, % | 0.97 (0.95-0.99) | <0.001 | 0.97 (0.95-0.99) | 0.002 | Included |  |
| LV LGE gray zone, g | 1.02 (1.00-1.03) | 0.022 | 1.03 (1.01-1.05) | 0.002 | Included |  |
| LV LGE core, g | 1.01 (0.99-1.02) | 0.12 | 1.02 (1.00-1.04) | 0.024 | - |  |
| LV LGE total, g | 1.01 (1.00-1.01) | 0.036 | 1.02 (1.01-1.03) | 0.002 | - |  |
| LAVI_max_, mL/m^2^ | 1.02 (1.01-1.03) | <0.001 | 1.00 (0.99-1.02) | 0.38 | - |  |
| LAVI_min_, mL/m^2^ | 1.02 (1.01-1.03) | <0.001 | 1.01 (0.99-1.02) | 0.18 | - |  |
| LAVI_preA_, mL/m^2^ | 1.02 (1.01-1.03) | <0.001 | 1.01 (0.99-1.02) | 0.87 | - |  |
| LA total emptying fraction, % | 0.97 (0.96-0.99) | <0.001 | 0.99 (0.98-1.00) | 0.27 | - |  |
| LA passive emptying fraction, % | 0.97 (0.95-0.98) | <0.001 | 0.98 (0.96-1.01) | 0.20 | - |  |
| LA active emptying fraction, % | 0.98 (0.97-0.99) | 0.007 | 0.99 (0.98-1.01) | 0.61 | - |  |
| Cine risk score | 4.13 (2.39-7.12) | <0.001 | 3.35 (1.88-5.98) | <0.001 | 2.27 (1.14-4.52) | 0.019 |

**Supplementary Figure 1**


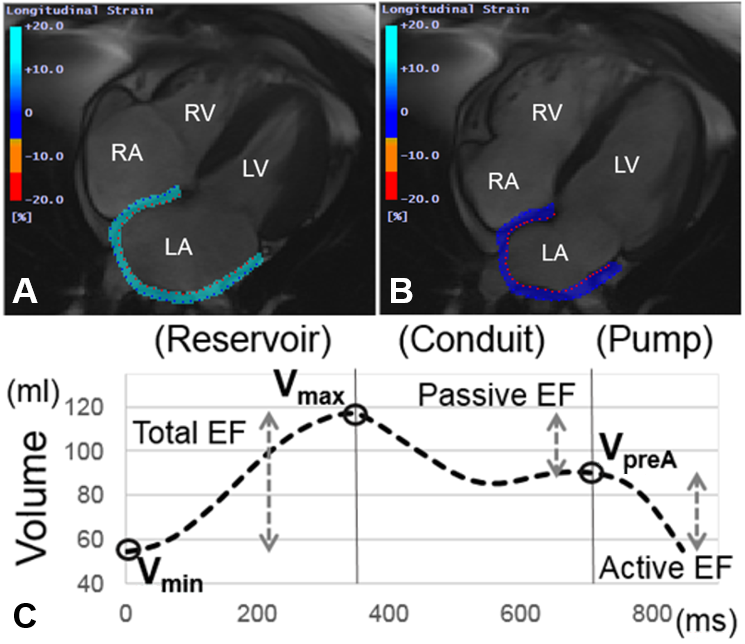


**Supplementary Figure 2**


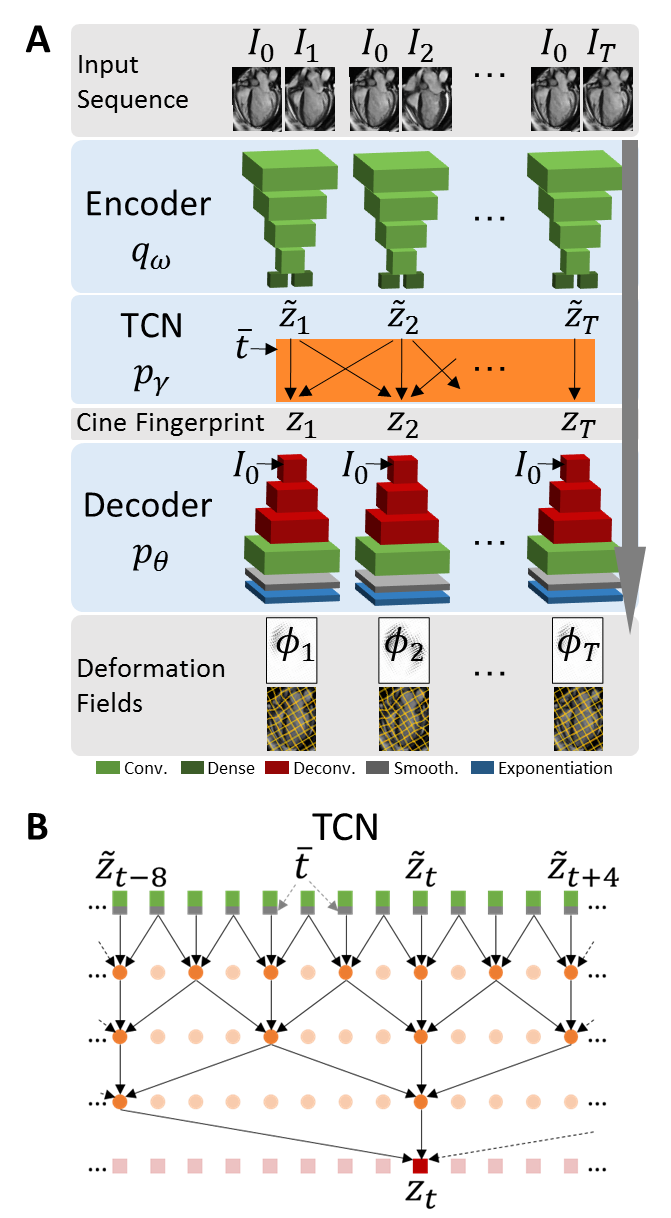

Supplement: Supplementary file 1 — Supplementary Information. [file 41598_2021_2111_MOESM1_ESM.docx]
